# Nurses’ strategies to provide emotional and practical support to the mothers of preterm infants in the neonatal intensive care unit: A systematic review and meta-analysis

**DOI:** 10.1177/17455057221104674

**Published:** 2022-06-23

**Authors:** Maryam Maleki, Abbas Mardani, Celia Harding, Mohammad Hasan Basirinezhad, Mojtaba Vaismoradi

**Affiliations:** 1 Pediatric and Neonatal Intensive Care Nursing Education Department, School of Nursing & Midwifery, Tehran University of Medical Sciences, Tehran, Iran; 2 Nursing Care Research Center, Department of Medical Surgical Nursing, School of Nursing & Midwifery, Iran University of Medical Sciences, Tehran, Iran; 3 Department of Language and Communication Science, City, University of London, London, UK; 4 Department of Epidemiology and Biostatistics, School of Public Health, Tehran University of Medical Sciences, Tehran, Iran; 5 Faculty of Nursing and Health Sciences, Nord University, Bodø, Norway; Faculty of Science and Health, Charles Sturt University, Orange NSW, Australia

**Keywords:** emotional support, infant, mother, neonatal intensive care unit, nursing, premature, systematic review

## Abstract

**Aim:**

To synthesize and integrate current international knowledge regarding nursing strategies for the provision of emotional and practical support to the mothers of preterm infants in the neonatal intensive care unit.

**Methods:**

A systematic review and meta-analysis was undertaken. Four English-language databases including EMBASE, PubMed (including MEDLINE), Scopus, and Web of Science were searched from January 2010 to October 2021. Original quantitative studies that were written in English and focused on nursing strategies for the provision of emotional and practical support to the mothers of preterm infants in the neonatal intensive care unit were included. Eligibility assessment, data extraction, and methodological quality appraisal were conducted independently by the review authors. A narrative synthesis of the review results and a meta-analysis were performed.

**Results:**

Twenty studies that were published from 2010 to 2021 were included in the review. Three categories concerning the review aims were identified: ‘nursing strategies related to mothers’ emotions and infant-mother attachment’, ‘nursing strategies related to mothers’ empowerment’, and ‘nursing strategies related to mothers’ participation in care process and support’. Eight interventional studies that reported mothers’ stress as the study outcome were entered into the meta-analysis. Interventions consisted of the educational programme, spiritual care, telenursing, parent support programme, skin-to-skin care, and guided family centred care. Significantly lower maternal stress was found in the intervention group compared with that of the control group (*g*: −1.06; 95% confidence interval: −1.64, −0.49; Z = 3.62, *p* < 0.001).

**Conclusion:**

This review identified and highlighted key nursing strategies used to provide emotional and practical support to the mothers of preterm infants in the neonatal intensive care unit. They included family centred care, skin-to-skin care, parent support and education programmes, interpersonal psychotherapy, spiritual care, newborn individualized developmental care and assessment programme, and telenursing.

## Introduction

Preterm birth as birth before 37 weeks of gestation is a global health problem.^1^ According to the World Health Organization (WHO),^2^ approximately 15 million preterm babies are born each year. Preterm infants require access to intensive neonatal care to survive.^3^ Therefore, preterm birth and hospitalization in the neonatal intensive care unit (NICU) create challenging situations for mothers.^1^ They encounter multiple stressors during the hospitalization of their preterm infants. Stress sources are the infant’s medical condition and his or her appearance, lower responsiveness to social interactions, abnormal breathing, limited availability of the infant, lack of information about the therapeutic regimen, and transition to parenthood.^4^ Moreover, NICU generates harmful stimuli such as excessive noise and light, interruption to sleep and excessive physical manipulation, all of which can impact negatively on an infant’s neurodevelopment.^3^ Furthermore, mothers experience a high degree of role confusion and negative emotions, such as frustration, stress, and anxiety due to unexpected hospitalization and uncertain prognosis of preterm infants. Therefore, mothers need for appropriate support.^5^ Nursing support can encourage the mothers of preterm infants to participate in care.^6^

Involvement of parents in the care of preterm infants and ensuring their understanding of rationales for nursing interventions can improve the outcomes of infant care, including quicker discharge to home.^7^ Development of strategies that reduce mothers’ stress and enable them to become engaged in preterm infant care leads to successful breastfeeding.^8^ Neonatal nurses along with other members of the neonatal healthcare team can reduce parents’ stress and help them develop infant care skills through education and role modelling.^9^ However, the identification of the range and type of support is difficult.^10,11^

Studies examining specific nursing strategies necessary for the parents of preterm or unwell term infants have focused on nurse–parent interactions and practical support to reduce parent stress through the provision of general advice and reassurance when gaining confidence with independent infant care.^12–14^ Neonatal nurses believe that they have a crucial role in helping parents interpret infant non-verbal signs and be confident in interacting with and caring for their infant through education and modelling to prepare for confident home parenting.^9,13,15^

Neonatal nurses undertake the provision of care through modelling and interaction for parents as well as the direct medical care of the infant within a stressful environment.^9^ Undertaking the implementation of such a wide range of professional strategies in a busy clinical setting can be hard, and parents have perceived inconsistent advice from nurses, poor communication, and fewer supported opportunities to develop independent infant care skills.^14–17^

Some studies have identified particular methods that nurses use to improve parent’s confidence with caring for their infant using essential strategies such as skin to skin.^18^ However, there is no systematic review and meta-analysis that investigated nursing strategies for provision of emotional and practical support to the mothers of preterm infants in the NICU. Therefore, this systematic review and meta-analysis aimed to synthesis and integrate current knowledge regarding nursing strategies for the provision of emotional and practical support to the mothers of preterm infants in the NICU. The review question was as follows: What strategies are used by nurses to provide emotional and practical support to the mothers of preterm infants in the NICU?

## Method

### Protocol and registration

The Preferred Reporting Items for Systematic Reviews and Meta-Analysis (PRISMA) guideline was used to structure this systematic review and subsequent meta-analysis (Supplemental File 1). The review protocol was registered with the International Prospective Register of Systematic Reviews (PROSPERO) and has the identifier CDR42020196361.

#### Definitions

For this review, it was felt important to focus specifically on the support mothers receive from nurses when developing skills to care for their preterm infant. We do not intend to negate the significance of fathers in neonatal care, and recognize the importance of involving fathers and partners in supporting and developing their own infants’ skills.^19^ However, as breastfeeding experiences are highly beneficial for infant development, it was felt that a close inspection of the international literature targeting the mothers of infants receiving care on a neonatal unit was worthy of specific investigation.^20^

We defined nursing strategies, which are used to provide emotional and practical support to the mothers of preterm infants in the NICU. Strategies which were sought included nurses directly supporting mothers implementing skin-to-skin (or kangaroo care (KC)) approaches, attachment, feeding skills, general cares for the infants, and emotional engagement.

### Eligibility criteria

Published quantitative studies including observational, randomized controlled trial (RCT), and quasi-experimental studies, in peer-reviewed scientific journals that focused on strategies used by nurses supporting both the emotional and practical needs of mothers in the NICU were included. Exclusion criteria were studies that reported on nurses who provided maternal support across the paediatric age range, that is, hospital wards, studies that gave inadequate information regarding nursing strategies for providing emotional and practical support to the mothers of preterm infants in the NICU, studies that focused solely on the fathers of preterm infants in the NICU, and studies that did not have a clear research methodology.

### Search methods for the identification of relevant studies

The following online bibliographic databases, Web of Science, PubMed (including MEDLINE), Scopus, EMBASE, and manual searching were utilized to identify papers from January 2010 to October 2021. The review authors used relevant literature and their knowledge to define the key words used, which were as follows: (nurs* AND support AND (preterm* OR premature* OR ‘low birthweight infant*’) AND mother* AND (neonate OR neonatal* OR ‘intensive care unit’ OR ‘critical care’ OR NICU)). The keywords were applied to develop search phrases and conduct the search using the Boolean method. All databases were searched using a similar set of terms, and all papers were entered into an Endnote library, with duplicates removed using both software and manual review. In addition, grey literature search and cross-referencing of bibliographies were performed.

### Study selection

Two review authors (M.M. and A.M.) performed independent investigations of relevant papers as part of the review process. Online conversations were conducted to share the search results and determine the subsequent steps of the study. During the search process the studies’ titles, abstracts, and full texts were retrieved and were screened by them. When there was a disagreement regarding the articles’ inclusion in the review, discussions were undertaken with a third author (M.V. or C.H.) to reach a consensus.

### Quality appraisal and risk of bias assessment

The quality of the included studies in the review was critically evaluated independently by the two review authors (M.M. and A.M.) in view of the methodological structure and presentation of results. The checklist entitled modified consolidated standards of reporting trials (CONSORT) was used for the appraisal of interventional studies. Accordingly, studies’ quality was categorized into four categories as follows: (1) scores of 70% of the highest scores on the checklist were rated high quality, (2) 40%–70% as moderate quality, (3) 20%–40% as low quality, and (4) <20% as very low quality.^21^ In addition, the quality of observational studies was appraised applying the modification of the Newcastle–Ottawa Quality Assessment Scale for non-randomized studies (NRS) in terms of selection, comparability, and outcomes. Studies with scores above 6 were considered as a high-quality study, 4–6 as a moderate quality study, and less than 4 as a low-quality study.^22^

The risk of bias of RCT studies was evaluated using the Cochrane Collaboration tool for evaluating the risk of bias for randomized clinical trials, which is categorized into low, high, and unclear risk of bias.^23^ For assessing risk of bias in quasi-experimental studies, Risk of Bias in Non-randomized Studies of Interventions (ROBINS-I) was used, which is categorized into low, moderate, serious, critical, and no information regarding risk of bias.^24^ Also, the risk of bias for cross-sectional studies adapted from the Newcastle–Ottawa Quality Assessment Scale was used for assessing the risk of bias of observational studies. Accordingly, the risk of bias was classified as low risk, probably low risk, probably high risk, and high risk of bias.^25^

### Data collection process and synthesis of results

Data extraction was performed through the use of a data extraction table developed by the authors. The table consisted of the first author surname, publication year, country of study origin, aim of the study, design and setting, sample size, intervention, measurement, and main findings. Next, the results of the included studies in the review were scrutinized and appropriate categories were created based on the study aims, and differences and similarities in their findings. The third author (M.V. or C.H.) approved the last version of the extracted date when there seemed to be any disagreement. In addition, the possibility of conducting a meta-analysis was assessed. Because of the great variety in the interventions and outcomes, eight interventional studies that reported mothers’ stress as the study outcome were entered into the meta-analysis.

### Data analysis

The mean and standard deviation of the maternal stress in the selected interventional studies that used Parental Stressor Scale: Neonatal Intensive Care Unit (PSS: NICU) for measuring stress was extracted by two review authors (A.M. and M.M.) independently and was analysed using STATA (Version 15, Stata Corporation, College Station, TX, USA). The Cochrane’s Q test was used to estimate heterogeneity among the included studies in the meta-analysis. A *p* value < 0.10 indicated significant heterogeneity and according to the *I*^2^ value, the heterogeneity degree was categorized into low (*I*^2^ < 25%), medium (25 ⩽ *I*^2^ < 50%), and high heterogeneity (*I*^2^ ⩾ 50%).^26^ A random-effects model was used if *p* < 0.10; otherwise, a fixed-effects model was adopted. The forest plot was used to summarize data in the included studies into the meta-analysis and the observed effects of individual studies along with the overall result. Meta-regression was performed to determine whether heterogeneity between the included studies could be attributed to covariates.^27^ The random-effects meta-regression model was conducted to investigate if the gestational age and educational level of mothers influenced the effect size of intervention. Statistical significance was set at *p* < 0.05.

## Results

### Search outcome and selection of studies

**Figure 1.**
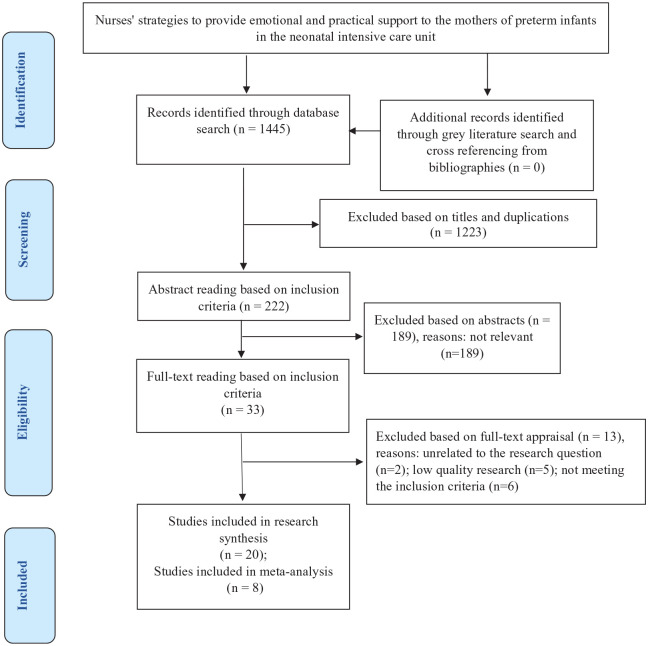
The preferred reporting items for systematic reviews and meta-analyses (PRISMA).

Results of the search process in the different databases are shown in Table 1. Accordingly, 1445 articles were retrieved using the predefined keywords. After removing irrelevant and duplicate titles and reading abstracts and full texts, 20 studies were selected for data analysis. No other studies were identified for inclusion in the grey literature search, manual search, and backtracking references. The study flow diagram according to the PRISMA is shown in Figure 1.

**Table 1.** Results of different phases of the search process.

Databases from 2010 to 2020	Total in each database	Title screening	Abstract reading	Full-text reading and appraisal
Web of Science	261	60	15	8
PubMed (including MEDLINE)	696	71	12	7
Scopus	299	58	5	4
Embase	189	33	1	1
Manual search/backtracking references	–	–	–	–
Total	1445	222	33	20

### Quality assessment and risk and bias

Evaluation of the methodological structure and quality of the presentation of results of the selected studies were performed during the full-text appraisal stage. Among 16 interventional studies, 9 studies had high quality and 7 studies were judged to have moderate quality (Supplemental Table 1). In addition, three observational studies had moderate quality and one had high quality (Supplemental Table 2).

The risk of bias results for nine randomized controlled studies is presented in Supplemental Figure 1. In terms of bias in random sequence generation, seven studies had a low risk of bias and two studies did not have insufficient information. Three studies were evaluated to have low risk of bias in the view of bias in allocation concealment and six studies failed to provide adequate information. In view of performance bias, five studies did not have sufficient information and four showed a low risk of bias. All studies except two, which had low risk of bias, did not present information regarding blinding of outcome assessment. Four studies had a high risk of bias due to incomplete outcome data (attrition bias). Furthermore, in five studies, the risk of bias in the selection of the reported result was unclear due to the lack of registered protocols to appraise pre-specified analysis plans and other studies had a low risk of bias.

The risk of bias results for seven NRS is provided in Supplemental Figure 2. In terms of bias due to confounding, three studies were judged to have low risk of bias, one moderate risk of bias, one serious risk of bias, and one critical risk of bias. The majority of studies in the view of bias in the selection of participants into the study (four studies), bias in the classification of interventions (five studies), bias due to deviations from intended interventions (four studies), and bias due to missing data (six studies) had a low risk of bias. In addition, three studies showed a low risk of bias, three studies did not have any information, and one had a moderate risk of bias in terms of bias in the measurement of the outcome. The risk of bias in the selection of the reported result was unclear in all of the studies due to the lack of registered protocols to appraise pre-specified analysis plans.

Furthermore, the results of risk of bias results for four observational studies are presented in the Supplemental Figure 3. All of the selected studies were judged to be low risk of bias in terms of the assessment of exposure and development of outcome of interest. In addition, in terms of the selection of cases and controls, three studies had probably high risk of bias and one had low risk of bias. Furthermore, two studies were evaluated to be low risk of bias and two probably high risk of bias in the view of control of prognostic variable.

### General characteristics of the selected studies

Table 2 shows an overview of selected studies (*n* = 20). All of them were published in English from 2012 to 2021. Six studies were from Iran,^31,35,42,44,45,47^ two from South Korea,^34,41^ two from Norway,^28,39^ two from the United States,^29,33^ one was from Canada,^32^ one from Japan,^38^ one from Denmark,^30^ one from Jordan,^46^ one from France,^40^ one from Italy,^36^ one from Sweden,^43^ and one from Turkey.^37^

**Table 2.** General characteristics of the selected studies included in data analysis and knowledge synthesis.

Ref citations, Country	Aim	Intervention	Measurement	Design/setting	Sample	Main findings
Wataker et al.,^28^ Norway	To describe the effects of an FC programme on maternal confidence and breastfeeding compared with mothers in a comparable NICU offering traditional care without such room facilities	Mothers in the FC group stayed in family rooms inside the NICU during their infant hospitalization	Researchers-made self-reporting questionnaire	A quasi-experimental/two level II NICUs	66 mothers: 36 in the FC group and 30 in the control group	Improving maternal confidence during the hospital stay and 3 months after discharge; increasing the level of empowerment; increasing breastfeeding
Schaffer et al.,^29^ USA	To examine the relationship between an 8-week relaxation guided imagery intervention on sleep quality and the association between sleep quality and maternal distress	Guided imagery was provided using three 20-min tracks. Participants listened to assigned songs once or more each day at any time of the day	PSQI; CES-D; STAI; Duke University of North Carolina Functional Social Support Questionnaire	Pre-experimental study: one-group pretest-posttest design/one NICU	19 mothers	Improvement of sleep quality
Weis et al.,^30^ Denmark	To examine the effect of the GFCC intervention, developed by the lead author, on parental stress in the NICU	The intervention group received GFCC intervention, which included helping parents control emotional stress and strengthen their ability to make decisions about caring for their infants	PSS: NICU; NPST	RCT/one level III NICU	74 parents: mothers *n* = 44; fathers *n* = 30 in the control group and 60 parents: mothers *n* = 31; fathers *n* = 29 in the intervention group	Parental stress and nurse-parent support did not significantly vary between two groups
Beheshtipour et al.,^31^ Iran	To explore the effect of the educational programme on the premature infants’ parental stress in the NICU	The intervention consisted of four training sessions with a booklet that was prepared with the content of the topics discussed in the training sessions	PSS: NICU	RCT/one NICU	29 mothers and 22 fathers in the intervention group and 29 mothers and 20 fathers in the control group	Reducing the premature infants’ maternal stress
Héon et al.,^32^ Canada	To examine the acceptability and feasibility of the breast milk expression education and support intervention in the mothers of preterm infants and study procedures	Breastfeeding expression training and supportive interventions were provided to the mothers of the intervention group	Breast milk expression diary; a questionnaire regarding acceptability of the educational and support components of the intervention	Pilot RCT/one NICU	14 mothers in the intervention group and 19 mothers in the control group	Improving breast milk production
Samra et al.,^33^ USA	To investigate the effect of SSC on stress perception between those mothers who provided SSC to their late-preterm born infants and those mothers who provided blanket holding	Mothers in the intervention group performed SSC at least 3 times a week with the duration of 50 min per session during their stay at NICU	PSS: NICU; SCRIP	RCT/one level III NICU	11 mothers in the control group: blanket holding group and 19 mothers in the intervention group: SSC group	SSC did not have a significant impact on maternal stress
Cho et al.,^34^ South Korea	To discover the effects of KC on the physiological functions of preterm infants, maternal–infant attachment, and maternal stress	KC was performed three times a week with 30-min duration a day for a total of 10 times	Maternal–infant attachment measurement tool; PSS	A quasi-experiment design; one general hospital	40 participants: 20 in the experimental group and 20 in the control group	Improving the maternal–infant attachment; reducing maternal stress
Peyrovi et al.,^35^ Iran	To determine the effect of empowerment programme on the perceived readiness for discharge of mothers of premature infants at the time of discharge	The intervention included a three-stage empowerment programme for the mothers of preterm infants	Parent discharge readiness questionnaire	A quasi-experimental study/two level II NICUs	80 mothers: 40 in the experimental and 40 in control groups	Improving mothers’ technical and emotional readiness to care for the premature infant
Sannino et al.,^36^ Italy	To evaluate the effectiveness of NIDCAP on mother’s support and infant development	Two NIDCAP-trained specialists assessed the infants’ current ability to organize and adjust subsystems in a caring interaction. Care recommendations then were developed to reduce stress and individual infants’ competence and development support	NPST	Non-randomized controlled study/one open space level III NICU	21 in NIDCAP group: 22 in standard care group	Improving the Nurse Parent Support; good sharing of mothers’ information with NICU staff; improvement of learning to take care of their child; improving mothers’ ability to cope with their child’s illness and long-term hospitalization
Alemdar et al.,^37^ Turkey	To explore the effect of spiritual care on the levels of stress in mothers with infants in a NICU	Spiritual care according to the interests of the mothers included reading the Quran, prayer, placed a cevşen-muska and a clipped evil-eye-talisman on the infant’s incubator	PSS-NICU	RCT/one second-level NICU	62 mothers: spiritual care group (*n* = 30) and control group (*n* = 32)	Reducing maternal stress
Shimizu and Mori,^38^ Japan	To investigate the maternal perceptions of family-centred support with hospitalized preterm infants, and its relationship to the collaboration between mothers and nurses in perinatal centres providing standard care	N/A	Neo-MPOC-20: Neo-EPS; an author-originated mother and infant questionnaire	Cross-sectional study/31 NICU of two types of perinatal centres	98 mothers whose infants were hospitalized in the NICU	Mothers’ perceptions were nearly always positive; the mean of three factors in the MPOC-20 was more than 5: consideration of parents’ feelings, ability to deal with specific needs, and coordination in dealing with situations; the mean of all factors of EPS also appeared positive; path analysis revealed that the relationship between mothers and nurses was linked to three factors related to the perinatal centres’ support: consideration of parents’ feelings, ability to deal with specific needs, and coordination in dealing with situations that interact with the provision of parent-friendly visual information
Tandberg et al.,^39^ Norway	To compare parent–infant closeness, parents’ perceptions of nursing support, and participation in medical rounds in single-family room (SFR) and an open bay (OB) NICUs	N/A	Parents recorded physical closeness prospectively in a closeness diary; questionnaire related to the parent participation and nursing support	A prospective survey / two NICU	33 infants from 29 families in the SFR unit and 31 infants from 29 families in the OB unit	Increasing the median presence, participation in decision-making and medical rounds and emotional support in mothers in SFR unit; increasing the nursing support including guidance, information, and emotional support from mothers in the SFR unit
Buil et al.,^40^ France	To assess the effects of a new skin-to-skin supported diagonal flexion (SDF) positioning on maternal stress, postpartum depression risk and skin-to-skin daily practice, in comparison with the usual KC in upright positioning, during the first weeks after very premature birth	N/A	PSS: NICU; EPDS	Case–control study/one level III NICU	34 mothers and their very preterm infants were assigned to one of the two Kangaroo Care positioning, either the upright (*n* = 17) or the SDF positioning (*n* = 17)	Reducing the risk of postpartum depression; lack of effect on maternal stress; a greater desire of mothers of the SDF group to perform KC longer
Heo and Oh,^41^ South Korea	To develop a parent participation improvement programme for parents in NICUs, and to evaluate its effects on parents’ partnerships with nurses, attachment to infants, and infants’ body weight	Participation improvement programme was provided for 2 weeks	Paediatric nurse–parent partnership scale; maternal attachment inventory	Mixed-methods (RCT section was considered)/one NICU	62 premature infants: intervention group (*n* = 30) and control group (*n* = 32) and their 132 parents: 66 mothers and 66 fathers	Improving the maternal-infant attachment and the mothers’ partnership with nurses; expressing the delight and excitement of parents being with their infants; more parental confidence; active participation care; feeling more responsibility
Jafarzadeh et al.,^42^ Iran	To examine the effect of telenursing on attachment and stress in the mothers of premature infants	Educational content including introduction, neonatal growth and development, treatment methods, infant relaxation, breastfeeding techniques, and soothing music were provided in the form of 10 unique telenursing codes and accessible in 24 h	PSS-NICU; MPA	RCT/one NICU	50 participants: 25 in the experimental group and 25 in the control group	Improving maternal postnatal attachment; reducing maternal stress
Månsson et al.,^43^ Sweden	To investigate the impact of an individualized neonatal parent support programme on parental stress	The support programme included parent-centred supportive communication based on their needs. Nurses provided educational content about home care, interaction with the neonate, and parental reactions	PSS-NICU	Quasi-experimental design/one NICU	Control group: 118 consisting of 60 mothers and 58 fathers; intervention group: 98 consisting of 49 mothers and 49 fathers	Reducing maternal stress
Moudi et al.,^44^ Iran	To evaluate the effectiveness of a care programme on the anxiety level of mothers with LAMP infants and to determine the effectiveness of the care programme on the level of anxiety of new mothers in the presence of social support	The care programme was implemented in four training sessions; at the end of each session, CDs and pamphlets were given to mothers about the content of the session	MSPSS; STAI-S	A quasi-experimental study/ one NICU	39 and 40 mothers in the intervention and control groups, respectively	Improving the perceived social support; reducing anxiety
Pouyan et al.,^45^ Iran	To identify the effects of an IPT-oriented childbirth education programme on maternal role adaptation and stress amongst the first-time child bearing women that babies were in NICU	The intervention group received two 60-min training sessions based on the IPT approach and one follow-up session by telephone call after discharge	PSS: NICU	RCT/one NICU	92 mothers: IPT group (*n* = 44) and control group (*n* = 43)	Reducing maternal stress; increasing maternal role adaptation
Al-Maghaireh et al.,^46^ Jordan	To examine the impact of an emotional support training programme on acute stress disorder level among the mothers of preterm infants admitted to an NICU	An emotional support programme was provided in two phases including the first phase of information and observation and the second phase of giving educational booklets to mothers	SASRQ	RCT/one level III NICU	50 mothers: 25, in the control group and 25 in the interventional group	Reducing maternal stress
Eskandari et al.,^47^ Iran	To describe the range and types of neonatal nursing support, as perceived by the mothers of preterm infants, and its association with mothers’ satisfaction of infant care in the NICU	N/A	Social Support Questionnaire; NIPS	A descriptive, correlational study/three NICUs	106 mothers	A moderate level of social support provided by nurses in terms of affirmational, concrete aid, affectional, and total social support; a significant relationship between nurses’ social support and mothers’ satisfaction with preterm infant care received in the NICUs

CES-D: Centre for Epidemiological Studies Depression Scale; EPDS: Edinburgh Postpartum Depression Scale; FC: family care; GFCC: guided family centred care; IPT: interpersonal psychotherapy; KC: kangaroo care; LAMP: late and moderate preterm; MPA: maternal postnatal attachment; MSPSS: multidimensional scale of perceived social support; Neo-EPS: Enabling Practice Scale in the NICU; Neo-MPOC 20: measure of process of care in the NICU; NICU: neonatal intensive care unit; NIDCAP: Newborn individualized developmental care and assessment program; NIPS: the neonatal instrument of parent satisfaction; NPST: nurse parent support tool; PSQI: Pittsburgh sleep quality index; PSS: Parental Stressor Scale; RCT: randomized controlled trial; SASRQ: Stanford Acute Stress Reaction Questionnaire; SCRIP: stability of the cardiorespiratory system in preterm infants; SSC: skin-to-skin care; STAI: State-Trait Anxiety Inventory; STAI-S: The State-Trait Anxiety Inventory.

Regarding the studies’ methodologies, nine studies used a RCT design,^15,30–33,37,41,42,46^ seven applied a quasi-experiment design,^28,29,34–36,43,44^ two used a cross-sectional,^38,47^ one used a case–control,^40^ and one used a prospective survey design.^39^

### Nursing strategies to provide emotional and practical support to the mothers of preterm infants in the NICU

**Figure 2.**
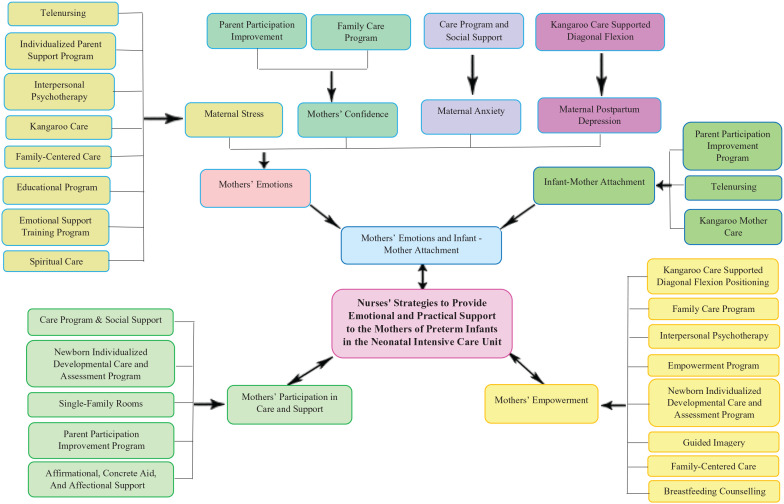
Nursing strategies used to provide emotional and practical support to the mothers of preterm infants in the neonatal intensive care unit.

After narrative analysis of the findings of the study, three categories concerning the nursing strategies used to provide emotional and practical support to the mothers of preterm infants in the NICU were identified: ‘nursing strategies related to mothers’ emotions and infant-mother attachment’, ‘nursing strategies related to mothers’ empowerment’, and ‘nursing strategies related to mothers’ participation in care process and support’. These categories with more details are presented in Figure 2.

### Nursing strategies related to mothers’ emotions and infant – mother attachment

This category discusses nursing strategies used related to the emotions of mothers of preterm infants including stress, confidence, excitement, postpartum depression, anxiety, and consideration of mothers’ feelings.

Seven RCT studies,^30,31,33,37,42,45,46^ two quasi-experimental studies,^34,43^ and one case-–control study^40^ investigated different nursing strategies on the stress level of mothers of preterm infants.

In the study by Alemdar et al.,^37^ spiritual care reduced mothers’ stress. In their study, mothers in the intervention group performed spiritual activities such as reading the Quran and prayers, and placed a cevşen-muska or a clipped evil-eye-talisman on the infant’s incubator. In a study by Cho et al.,^34^ mothers who used kangaroo mother care (KMC) three times a week experienced less stress and improved infant–mother attachment.

A training programme using telenursing were designed in a RCT study. The educational content included introducing the section, features of the premature baby, treatment methods, relaxation techniques such as massage, KMC, breastfeeding, and soothing music that mothers could access to each content by dialling the assigned codes. The results showed a reduction in maternal stress in the intervention group. In addition, there was a greater improvement in the infant–mother attachment.^42^ Another study found that an individualized parent support programme reduced maternal stress. The support programme designed by this study focused on family centred care (FCC) and person-centred communication, and teaching infant traits, meeting infant needs, how to interact with infants, parental responses, and home care.^43^

A RCT reported that mothers who received interpersonal psychotherapy (IPT) had less stress. IPT delivered during two training sessions and contents consisted of goal setting and training on infant development, barriers to communication with infants, motherhood and maternal roles, postpartum depression symptoms, coping strategies, problem-solving skills, and infant communication skills. The third session of the intervention was telephone follow-up.^45^

An emotional support training programme for mothers was conducted by Al-Maghaireh et al.^46^ This programme focused on face-to-face training on familiarity with the ward physical environment, equipment, terminology, features of preterm infants, procedures, care methods, KMC, and defence mechanisms along with the delivery of an educational booklet. The findings showed that mothers in the intervention group experienced low levels of stress.

In another study, a training programme with the content of familiarity with the physical environment of the ward and equipment, providing information about the general, current, and future condition of neonates, teaching how to support the spouse and solving parents’ problems were developed. In addition, they provided an educational booklet on the topics of the training sessions to the mothers. The results of their study showed that the training programme had a significant effect on reducing maternal stress.^31^

Interventions in three studies had no effect on maternal stress. Two studied evaluated the effect of skin-to-skin care (SSC). Accordingly, the results of a case–control study showed that KC supported diagonal flexion (SDF) positioning for a month in comparison with the usual KC in upright positioning had no effect on maternal stress.^40^ In addition, in another study, SSC was performed for 50 min three times a week for mothers and the results showed that SSC had no effect on maternal stress.^33^ Also, Weis et al.^30^ implemented an FCC programme based on person-centred nurse–parent communication and reported that the programme had no effect on maternal stress.

The results of two studies showed the effect of nursing interventions on the improvement of mothers’ confidence. The programme to improve parental involvement in the Heo and Oh’s^41^ study included three phases of individual interaction: the pre-participation phase including familiarity with the ward environment and equipment, infant characteristics, and the active participation phase including KMC, bathing, breastfeeding, singing, and conversation. Mothers who participated in this programme had higher confidence and felt excited and happy to be with their infants. Furthermore, improvement in mother–infant attachment was another result of this study. In another study, mothers receiving the family care (FC) programme reported higher confidence. Mothers in the FC group stayed inside the NICU in family rooms with special facilities during the infant’s hospitalization.^28^

The results of a case–control study showed that skin-to-skin SDF positioning can reduce the risk of maternal postpartum depression.^40^ In a quasi-experimental study, a care programme was designed to educate mothers. The content of this four-session training programme was about familiarity with the environment, their infant’s condition, hand hygiene, breastfeeding, calming crying and colic, and post-discharge care. A reduction in maternal anxiety in the intervention group was observed.^44^ Another cross-sectional study reported that mothers’ perceptions of FCC, including KMC, mothers’ participation in activities such as feeding, bathing, and changing diapers were positive, and mothers felt that nurses paid enough attention to their feelings.^38^

### Nursing strategies related to mothers’ empowerment

This category describes nursing strategies related to mothers’ empowerment including learning to care for the infants, ability of mothers to cope with illness and hospitalization of their infants, improvement of maternal role adaptation, mothers’ desire to perform long KMC, enhancement of the level of empowerment, emotional and practical readiness of mothers to care, increase of breast milk production, ability to meet special needs, coordination in dealing with situations, and improving the quality of mothers’ sleep.

In the study by Sannino et al.,^36^ two Newborn Individualized Developmental Care and Assessment Programme (NIDCAP)-trained specialists assessed infants’ current abilities to organize and adjust subsystems in a caring interaction. Care recommendations were developed to reduce stress and individual infants’ competence and support parents and nurses to implement the programme to the time of discharge. The results of this quasi-experimental study showed that mothers’ abilities to learn to care for their infants and cope with illness and hospitalization of their infants improved.

Another study investigated the effect of IPT and findings revealed that maternal role adaptation increased in mothers who received IPT.^45^ Furthermore, practicing skin-to-skin SDF positioning in comparison with usual KC in upright position for a month were more likely to do long-term KMC.^40^ In addition, implementing the FC programme had a significant effect on the improvement of breastfeeding and mothers’ level of empowerment.^28^

A three-session empowerment programme for the mothers of preterm infants with an educational booklet was developed in a quasi-experimental study. This programme included familiarity with the ward, infant’s characteristics, sleep patterns, interaction with the infant, nutrition, umbilical cord care, active involvement of the mother in infant care, follow-up screening tests, vaccinations, medications, and how to communicate with NICU staff if needed. The results of this study showed that the empowerment programme improved mothers’ emotional and technical readiness to care for their infants.^35^

In a RCT study, breastfeeding counselling was provided to mothers assigning in the intervention group. Counselling topics consisted of breastfeeding expression training and supportive interventions. Increased breast milk production was reported by the mothers in the intervention group.^32^ Also, FCC was directly associated with mothers’ abilities to cope with special needs and situations.^38^

In a pre-experimental study, Schaffer et al.^29^ examined the effect of an 8-week guided imagery (GI) intervention on maternal sleep quality. Participants listened to dedicated songs every day and at any time of the day. Improvement of sleep quality in mothers receiving the intervention was one main result of this study.

### Nursing strategies related to mothers’ participation in the care process and support

This category describes nursing strategies related to mothers’ participation in the care process and its outcomes. Development and implementation of the participation improvement programme by Heo and Oh^41^ improved the active participation of mothers in caring for their infants and increased their sense of responsibility. Also, provision of care according to the NIDCAP programme increased nurses’ support for parents and resulted in a good sharing of mothers’ information with nurses.^36^ The care programme developed by Moudi et al.^44^ increased nurses’ support for mothers.

A cross-sectional study showed that mothers perceived nursing support in terms of affirmational, concrete aid, and affectional in an acceptable level and the perceived nursing support was significantly associated with mothers’ satisfaction with preterm infant care in the NICU.^47^ In another study, the amount of support provided by nurses in the NICU with single-family rooms (SFRs) was compared with the amount of support provided by nurses in an open bay (OB) NICU. The results showed that the presence of mothers in the SFR group had positive consequences including enhancement of the median presence of mothers with their infants, increase of their participation in decision-making and medical rounds, and increase of the nursing support including guidance, information, and emotional support.^39^

### Results of meta-analysis for mothers’ stress

**Figure 3.**
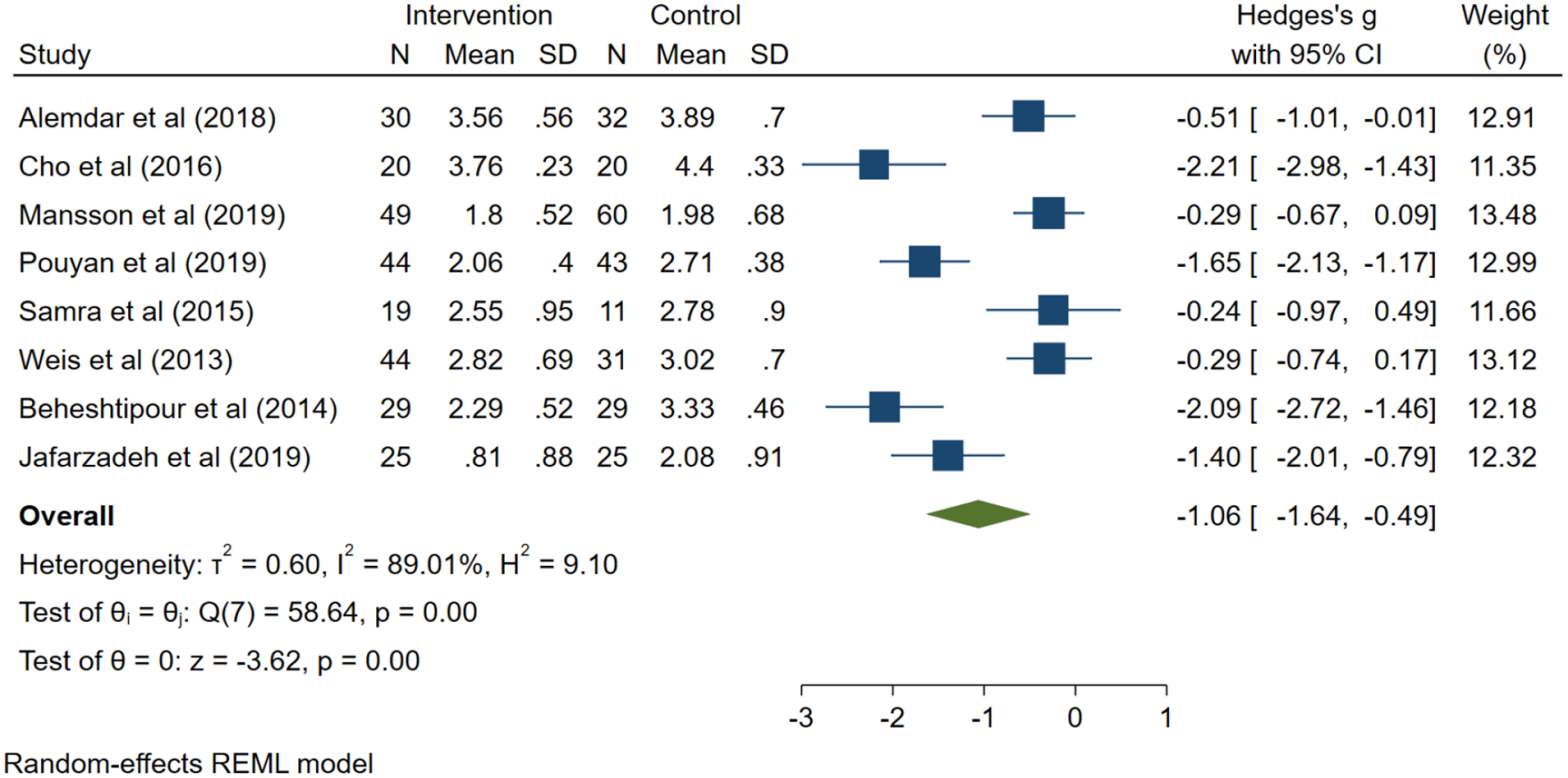
Meta-analysis of the mothers’ stress based on the random-effect model.

Eight studies^30,31,33,34,37,42,43,45^ consisting of six RCTs and two quasi-experimental studies reported mothers’ stress as the study outcome with the same scale. Interventions were including educational programme, spiritual care, telenursing, parent support programme, SSC, and guided family centred care. Since overall heterogeneity was observed in the studies (*I*^2^ *=* 89.01%, *p* < 0.001), a random-effects model was applied. A total of 511 participants were included. Significantly lower maternal stress was found in the interventional group compared with that of the control group (*g*: −1.06; 95% confidence interval: −1.64, −0.49; Z = 3.62; *p* < 0.001; Figure 3).

The random-effects meta-regression showed that the mean gestational age in the control group (*p* = 0.04) and the intervention group (*p* = 0.03) affected the *I*^2^ value and reduced the *I*^2^ value from 89.01% to 79%.

## Discussion

To the best of our knowledge, the present study is the first systematic review and meta-analysis that synthesized the findings of quantitative studies and integrated knowledge regarding nursing strategies used to provide emotional and practical support to the mothers of preterm infants in the NICU. The findings of this review showed that various strategies were used by nurses to provide support for mothers’ emotions and mother–infant attachment, mothers’ empowerment, and mothers’ participation in the care process and support in NICUs.

Perceived maternal stress was the most frequently measured outcome of all maternal emotional outcomes. This is not unexpected because the literature has reported high levels of stress for parents, particularly for mothers with preterm infants in the NICU. Parents are facing a circumstance in which their vulnerable infant’s life is being threatened and also they are unfamiliar with the technical environment, the equipment around their infant, and the intercommunication between NICU nurses and medical staff.^48^ Therefore, nursing interventions for the reduction of mothers’ stress are very important.^49^ The findings showed that using different strategies by nurses including spiritual care, KMC, telenursing, individualized parent support programme using FCC and person-centred communication, IPT, and training programmes can reduce stress level of preterm infants’ mothers in NICUs. Similarly, a systematic review found that interventions such as FC education and psychotherapy reduced the distressing symptoms of parents with neonates admitted to the NICU.^50^ The review conducted by Ding et al.^51^ reported that parents of preterm infants in the NICU who received FCC interventions experienced lower stress. FCC interventions in this review were most related to training, giving information, and involving parents in the care process and decision-making.^51^ In addition, the findings of another systematic review suggested that applying KMC in preterm infants contributed to the decrease of maternal stress.^52^ Due to the high prevalence of maternal stress when their preterm infants are admitted to the NICU, including strategies in the care programme to decrease their stress is important.

According to our findings, providing parent participation improvement and FC programmes improve confidence, care programme and social support improve anxiety, and skin-to-skin SDF positioning reduces the risk of postpartum depression in mothers of preterm infants in the NICU. A systematic review on qualitative studies concluded that nurses with the implementation of KMC are able to assist with preparing and guiding parents, and promote their self-confidence and ability in their parental roles.^48^ The systematic review and meta-analysis conducted by Scime et al.^53^ demonstrated that SSC had significant small protective effect on postpartum depression among the mothers of preterm or low birthweight infants. SSC could prevent the onset of postpartum depression by optimizing maternal physiology through stimulating oxytocin and promoting attachment.^54^ Furthermore, it has been shown that providing FCC and parent support programmes in the NICU help parents feel less stressed and more confident in their infant care.^55^ Moreover, reduction of the level of anxiety and improving maternal confidence in infant care is the successful outcome of programmes such as SSC, Mother–Infant Transaction Programme (MITP), Creating Opportunities for the Parent Empowerment Programme (COPE), Video Interaction Guide (VIG), and partnership care programme.^56^

The infant–mother attachment disorder can have adverse consequences for infants such as separation anxiety and failure to thrive, and for parents such as depression and anxiety.^56^ According to our review, interventions including KMC, telenursing, and the participation improvement programme improved maternal–infant attachment. Similarly, Salehi et al.^56^ in a systematic review stated that Guided Partnership (GP), SSC, MITP, and COPE were effective in increasing maternal and infant attachment and maternal engagement. In addition, MITP and GP increased breastfeeding skills in the mothers of preterm infants. It seems KMC and SSC have physical and biological benefits to neonates, as well as to their mothers and they can facilitate infant–mother attachment.^57^

Empowering parents as primary caregivers is essential to improve the well-being and development of infants. The findings of the present systematic review indicated that nursing interventions including the NIDCAP, IPT, SDF positioning, the FC programme, empowerment programme, breastfeeding counselling, and GI were useful for improving mothers’ empowerment. Similarly, the preterm infants’ mothers in a qualitative study reported that providing NIDCAP care is accompanied by an increase in their empowerment with knowledge and confidence.^58^ The IPT can empower mothers by the successful role transition and improve their self-efficacy through increasing the mother–infant attachment and compliance of the mother’s role.^59,60^ The SDF positioning creates more comfortable for mothers and can lead to prolonging this moment and offers an opportunity for both infant and mother to find each other and intercommunication in synchrony.^61,62^ In FCC, nurses value parents’ concerns and actively attempt their engagement. They empower parents by instilling a fundamental understanding and skills while providing possibilities for parents to participate in their infant’s care. Indeed in this model, parents are valued as a member of their infant’s care team and are appreciated for their significant and unique position in the care team.^55,63^ NICU nurses can help parents and play an important role in the development of early mother–infant relationships.^35^ In addition, they have the most contact with the mothers of infants and their families, so they are in an ideal position to participate in the mothers’ empowerment process using research findings such as breastfeeding counselling and empowerment programme.^64–66^

Our systematic review showed that the participation improvement programme, the NIDCAP programme, the care programme, and SFR improved mothers’ participation in the care process and support. The results of a systematic review showed that parents with preterm infants admitted to SFR NICUs experienced low levels of NICU-related stress and parental presence, empowerment, participation in care, and satisfaction increased.^67^ Another systematic review reported an increase in exclusive breastfeeding at discharge and a lower incidence of sepsis in SFR NICUs.^68^ Parental involvement in infant care leads to long-term positive outcomes for both infants and parents themselves.^67^ Therefore, the implementation of nursing interventions and strategies evidenced in this review is recommended to strengthen parental participant in care.

## Implications for clinical practice

Our study extended the international knowledge about nursing strategies used to provide emotional and practical support to the mothers of preterm infants in the NICU. The findings of this systematic review and meta-analysis can be used by nurses and healthcare leaders to devise appropriate strategies such as spiritual care, SSC, specific educational, support, and empowerment programmes, and NIDCAP for providing optimal emotional support to the mothers of preterm infants in the NICU. The parents of preterm infants should be empowered and encouraged to participate in the infant care process in the NICU.

## Limitations

Only articles published in English were included in this review. In addition, given the heterogeneity in the outcomes and interventions of the studies included in this systematic review, a meta-analysis for all outcomes except for maternal stress could not be conducted. Furthermore, studies in which mothers were participants along with fathers if a separate analysis for mothers was not available were excluded. The limited number of studies on some interventions such as IPT, GI, and SDF in the mothers of preterm infants made it difficult to draw strong conclusions for nursing practice. In terms of risk of bias, most RCTs failed to provide adequate information regarding performance bias, bias in allocation concealment, and blinding of outcome assessment. Also, most RCTs and all NRS did not provide sufficient information about bias selection of reported results, which might have reduced reliance on the studies’ results.

## Conclusion

In this systematic review and meta-analysis, nursing strategies for the provision of emotional and practical support to the mothers of preterm infants in the NICUs were presented. Different strategies suggested by this review such as FCC, SSC, parent support and education programmes, IPT, spiritual care, NIDCAP, and telenursing can be applied by nurses in NICUs to provide appropriate emotional and practical support to the mothers of preterm infants. In addition, it is recommended to start educational and support programmes for parents with preterm infants admitted to the NICU so that they are empowered to collaborate with healthcare providers in caring for their infants.

By recognizing omissions in the body of international literature, this systematic review and meta-analysis highlighted some opportunities for future research. Researchers have the opportunity to design, test, and adapt interventions and strategies in many areas of the world that currently has limiting research on NICU parental support. Future studies ideally can evaluate the effectiveness of interventions and strategies across the lengthy post NICU discharge period. Finally, this review focused on the mothers of preterm infants, which future studies should include also fathers of this group.

## Supplemental Material

sj-docx-1-whe-10.1177_17455057221104674 – Supplemental material for Nurses’ strategies to provide emotional and practical support to the mothers of preterm infants in the neonatal intensive care unit: A systematic review and meta-analysis Click here for additional data file. Supplemental material, sj-docx-1-whe-10.1177_17455057221104674 for Nurses’ strategies to provide emotional and practical support to the mothers of preterm infants in the neonatal intensive care unit: A systematic review and meta-analysis by Maryam Maleki, Abbas Mardani, Celia Harding, Mohammad Hasan Basirinezhad and Mojtaba Vaismoradi in Women’s Health

sj-docx-2-whe-10.1177_17455057221104674 – Supplemental material for Nurses’ strategies to provide emotional and practical support to the mothers of preterm infants in the neonatal intensive care unit: A systematic review and meta-analysis Click here for additional data file. Supplemental material, sj-docx-2-whe-10.1177_17455057221104674 for Nurses’ strategies to provide emotional and practical support to the mothers of preterm infants in the neonatal intensive care unit: A systematic review and meta-analysis by Maryam Maleki, Abbas Mardani, Celia Harding, Mohammad Hasan Basirinezhad and Mojtaba Vaismoradi in Women’s Health
