# Supplementary material for: Nurses’ strategies to provide emotional and practical support to the mothers of preterm infants in the neonatal intensive care unit: A systematic review and meta-analysis
Source: Womens Health (Lond). 2022 Jun 23;18:17455057221104674. doi: 10.1177/17455057221104674 (PMC9234836; doi:10.1177/17455057221104674)
Supplement: sj-docx-2-whe-10.1177_17455057221104674 – Supplemental material for Nurses’ strategies to provide emotional and practical support to the mothers of preterm infants in the neonatal intensive care unit: A systematic review and meta-analysis [file sj-docx-2-whe-10.1177_17455057221104674.docx]

**Supplementary file 2**

**Table 1.** Quality assessment of the selected studies using the CONSORT Assessment Scale for interventional studies.

| **Author, year** | **Code** | **Methods** | | | | | | | | | | | | | | | | |
| --- | --- | --- | --- | --- | --- | --- | --- | --- | --- | --- | --- | --- | --- | --- | --- | --- | --- | --- |
|  |  | **Trial design** | | **Participants** | | **Interventions** | **Outcomes** | | **Sample size** | | **Randomization** | | **Allocation concealment mechanism** | **Implementation** | **Blinding** | | **Statistical methods** | |
|  |  | a | b | a | b |  | a | b | a | b | a | b |  |  | a | b | a | b |
| Wataker et al, 2012 (44) | 17 |  |  | * | * | * | * | * |  |  |  |  |  |  | * | * | * |  |
| Schaffer et al, 2013 (46) | 29 | * | * | * | * | * | * | * |  |  |  |  |  |  |  | * | * |  |
| Weis et al, 2013 (49) | 24 | * | * | * | * | * | * | * |  |  |  |  | * |  | * | * | * |  |
| Beheshtipour et al, 2014 (36) | 28 | * | * | * | * | * | * | * | * | * | * | * |  |  | * | * | * |  |
| Héon et al, 2014 (47) | 21 | * | * | * | * | * | * | * | * | * | * | * | * |  | * | * |  |  |
| Samra et al, 2015 (45) | 18 | * | * | * | * | * | * | * | * | * | * | * | * | * | * | * | * |  |
| Cho et al, 2016 (41) | 3 | * | * | * | * | * | * | * | * | * |  |  |  |  | * | * | * | * |
| Peyrovi et al, 2016 (39) | 19 | * | * | * | * | * | * | * |  |  |  |  |  |  | * | * | * |  |
| Sannino et al, 2016 (52) | 9 | * | * | * | * | * | * | * | * | * |  |  |  |  | * | * | * |  |
| Alemdar et al, 2018 (54) | 1 | * | * | * | * | * | * | * | * | * |  |  |  |  | * | * | * |  |
| Heo and Oh, 2019 (42) | 4 | * | * | * | * | * | * | * | * | * | * |  |  |  | * | * | * | * |
| Jafarzadeh et al, 2019 (37) | 5 |  | * | * | * | * | * | * | * | * | * |  |  |  | * | * | * |  |
| Mansson et al, 2019 (53) | 8 | * | * | * | * | * | * | * |  |  |  |  |  |  | * | * | * |  |
| Moudi et al, 2019 (38) | 20 | * | * | * | * | * | * | * | * | * |  |  |  |  | * | * | * |  |
| Pouyan et al, 2019 (40) | 13 |  | * | * | * | * | * | * | * | * |  | * |  |  | * | * |  |  |
| Al-Maghaireh et al, 2020 (50) | 16 | * | * | * | * | * | * | * | * | * |  |  |  |  | * | * | * |  |

| **Author, year** | **Results** | | | | | | | | | | **Total score** | **Quality** |
| --- | --- | --- | --- | --- | --- | --- | --- | --- | --- | --- | --- | --- |
|  | **Participant flow (a diagram is strongly recommended)** | | **Recruitment** | | **Baseline data** | **Numbers analyzed** | **Outcomes and estimation** | | **Ancillary analyses** | **Harms** |  |  |
|  | a | b | a | b |  |  | a | b |  |  |  |  |
| Wataker et al, 2012 (44) | * | * |  | * | * | * | * |  |  |  | 14 | Moderate |
| Schaffer et al, 2013 (46) | * | * | * | * | * | * |  |  |  |  | 15 | Moderate |
| Weis et al, 2013 (49) | * | * | * | * | * | * | * |  | * |  | 19 | High |
| Beheshtipour et al, 2014 (36) | * | * | * | * | * | * | * | * |  |  | 22 | High |
| Héon et al, 2014 (47) | * | * | * | * | * | * | * |  |  | * | 22 | High |
| Samra et al, 2015 (45) | * | * | * | * | * | * | * |  | * |  | 24 | High |
| Cho et al, 2016 (41) | * | * | * | * | * | * | * |  |  |  | 20 | High |
| Peyrovi et al, 2016 (39) | * | * | * | * | * | * | * |  |  |  | 17 | Moderate |
| Sannino et al, 2016 (52) | * | * | * | * | * | * | * |  |  |  | 19 | High |
| Alemdar et al, 2018 (54) | * | * | * | * | * | * | * |  |  |  | 19 | High |
| Heo and Oh, 2019 (42) | * | * | * | * | * | * | * |  |  |  | 21 | High |
| Jafarzadeh et al, 2019 (37) |  |  | * | * | * | * | * |  |  |  | 17 | Moderate |
| Mansson et al, 2019 (53) |  | * | * |  | * | * | * |  |  |  | 15 | Moderate |
| Moudi et al, 2019 (38) | * | * | * | * | * | * | * |  | * |  | 20 | High |
| Pouyan et al, 2019 (40) | * | * |  |  | * | * | * |  |  |  | 16 | Moderate |
| Al-Maghaireh et al, 2020 (50) |  | * |  | * | * | * | * |  |  |  | 17 | Moderate |

**Table 2.** Quality assessment of the selected studies using the Newcastle–Ottawa Quality Assessment Scale for cross-sectional studies.

|  | **Selection** | | | | **Comparability** | **Outcome** | | **Total score** | **Quality** |
| --- | --- | --- | --- | --- | --- | --- | --- | --- | --- |
| **Author, year** | **Representativeness of the samples** | **Sample size** | **Non-responders** | **Ascertainment of the exposure** | **A: study controls for age and/or BMI**  **B: control for any additional factor** | **Assessment of the outcome**  **a) Independent blind assessment**  **b) Record linkage**  **c) Self report** | **Statistical test** |  |  |
| Shimizu et al, 2018 (48) | * | * | * | * |  | * | * | 6 | Moderate (Satisfactory) |
| Tandberg et al, 2018 (43) | * |  | * | * | * | * | * | 6 | Moderate (Satisfactory) |
| Buil et al, 2019 (51) | * |  | * | * | ** | ** | * | 8 | High |
| Eskandari et al, 2021 (35) |  | * | * | * |  | * | * | 5 | Moderate (Satisfactory) |

**Figure 1.** Risk of bias in randomized controlled studies.

A:

| Author, year | A | B | C | D | E | F |
| --- | --- | --- | --- | --- | --- | --- |
| Weis et al, 2013 (49) | 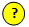 | 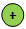 | 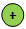 | 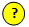 | 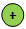 | 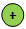 |
| Beheshtipour et al, 2014 (36) | 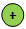 | 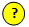 | 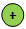 | 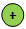 | 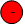 | 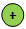 |
| Héon et al, 2014 (47) | 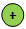 | 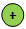 | 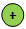 | 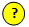 | 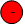 | 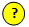 |
| Samra et al, 2015 (45) | 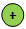 | 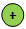 | 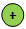 | 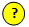 | 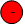 | 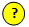 |
| Alemdar et al, 2018 (54) | 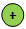 | 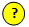 | 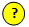 | 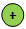 | 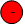 | 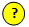 |
| Heo and Oh, 2019 (42) | 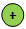 | 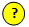 | 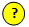 | 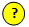 | 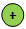 | 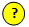 |
| Jafarzadeh et al, 2019 (37) | 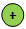 | 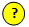 | 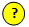 | 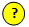 | 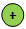 | 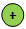 |
| Pouyan et al, 2019 (40) | 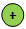 | 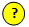 | 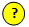 | 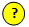 | 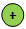 | 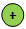 |
| Al-Maghaireh et al, 2020 (50) | 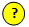 | 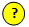 | 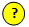 | 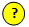 | 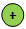 | 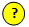 |
| 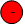 Yes (high risk of bias) 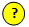 Unclear 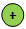 No (low risk of bias)  (A) Bias in random sequence generation (selection bias)  (B) Bias in allocation concealment (selection bias)  (C) Bias in the blinding of participants and personnel (performance bias)  (D) Bias in the blinding of outcome assessment (detection bias)  (E) Bias in incomplete outcome data (attrition bias)  (F) Bias in selective outcome reporting (reporting bias) | | | | | | |

B:

**Figure 2.** Risk of bias in non-randomized clinical studies.

A:

| Author, year | A | B | C | D | E | F | G |
| --- | --- | --- | --- | --- | --- | --- | --- |
| Wataker et al, 2012 (44) |  | 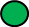 | 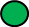 | 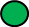 | 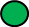 |  |  |
| Schaffer et al, 2013 (46) |  |  |  |  | 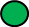 |  |  |
| Cho et al, 2016 (41) |  |  | 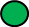 | 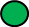 | 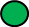 | 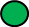 |  |
| Peyrovi et al, 2016 (39) | 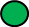 | 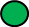 | 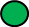 | 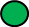 | 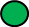 | 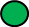 |  |
| Sannino et al, 2016 (52) | 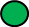 |  |  |  | 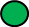 |  |  |
| Mansson et al, 2019 (53) |  | 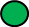 | 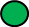 | 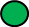 |  | 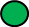 |  |
| Moudi et al, 2019 (38) | 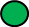 | 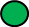 | 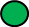 |  | 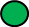 |  |  |
| 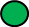  Low risk of bias Moderate risk of bias  Serious risk of bias Critical risk of bias  No information  (A) Bias due to confounding (confounding)  (B) Bias in selection of participants into the study (selection bias)  (C) Bias in classification of interventions (information bias)  (D) Bias due to deviations from intended interventions (confounding)  (E) Bias due to missing data (selection bias)  (F) Bias in measurement of the outcome (information bias)  (G) Bias in the selection of the reported result (reporting bias) | | | | | | | |

B:

**Figure 3.** Risk of bias in cross-sectional studies.

A:

| Author, year | Bias in the assessment of exposure | Bias in the development of outcome of interest in case and controls | Bias in the selection of cases | Bias in the selection of controls | Bias in the control of prognostic variable |
| --- | --- | --- | --- | --- | --- |
| Shimizu et al, 2018 (48) |  |  |  |  |  |
| Tandberg et al, 2018 (43) |  |  |  |  |  |
| Buil et al, 2019 (51) |  |  |  |  |  |
| Eskandari et al, 2021 (35) |  |  |  |  |  |
| Definitely No (low risk of bias) Probably no (Probably low risk of bias)  Definitely yes (high risk of bias) Probably Yes (Probably high risk of bias) | | | | | |

B:
